# Predictors of Vitamin D Insufficiency in Children and Adolescents With Alopecia Areata

**DOI:** 10.7759/cureus.22934

**Published:** 2022-03-07

**Authors:** Rachel K Lim, Leslie Castelo-Soccio, Elana Putterman, Abrar A Qureshi, Eunyoung Cho

**Affiliations:** 1 Department of Dermatology, Warren Alpert Medical School of Brown University, Providence, USA; 2 Division of Pediatrics, Section of Dermatology, The Children’s Hospital of Philadelphia, University of Pennsylvania Perelman School of Medicine, Philadelphia, USA; 3 Department of Dermatology, University of Massachusetts Medical School, Worcester, USA; 4 Department of Dermatology, Warren Alpert Medical School, Brown University, Providence, USA; 5 Department of Epidemiology, School of Public Health, Brown University, Providence, USA

**Keywords:** hair loss, public health, pediatric dermatology, dermatology, alopecia areata, vitamin d, nutrition

## Abstract

Introduction: Limited data regarding predictors of vitamin D deficiency in US children exist. We aimed to identify predictors of vitamin D insufficiency among children with alopecia areata.

Methods: The medical records of 439 pediatric patients diagnosed with alopecia areata (AA) between January 2015 and December 2017 were reviewed. Those with 25-hydroxyvitamin D levels and no documented vitamin supplementation, chronic illness, or other autoimmune comorbidities other than AA were included. Demographic data, Fitzpatrick skin type, and the month of blood collection were recorded. Monthly UV index information from Philadelphia, PA corresponding to the month of blood collection was also collected.

Results: Within our cohort, 60.4% of patients had insufficient vitamin D levels, of which 38.2% were deficient. The mean age was nine years old. In multivariate analyses, higher Fitzpatrick skin type, non-summer season, and non-White race were associated with vitamin D insufficiency, while the monthly UV index was inversely associated.

Discussion/Conclusion: Higher Fitzpatrick skin type, non-summer season, and non-White race may be associated with vitamin D insufficiency in US pediatric patients. Larger studies are warranted to replicate our findings and fully evaluate predictors of pediatric vitamin D deficiency in the US.

## Introduction

Vitamin D is a fat-soluble vitamin important for a variety of cell functions. In particular, vitamin D plays an important role in calcium absorption and bone health. Vitamin D is primarily made in the skin after exposure to ultraviolet (UV) radiation, and very little is derived from dietary sources [1]. Vitamin D deficiency can impair calcium absorption, increase bone turnover, and decrease bone density. In severe cases, vitamin D deficiency can cause rickets, the failure of mineralization of growing bones and cartilage in children [2]. Vitamin D deficiency also has been associated with increased risks of certain autoimmune diseases, psychiatric disorders, and cancers [2].

Previously identified predictors of vitamin D in both pediatric and adult populations include age, gender, skin color, and socioeconomic status. Nevertheless, limited data exist on predictors of vitamin D in children, especially in the United States [3-5]. Therefore, we aim to identify predictors of vitamin D insufficiency among pediatric patients with alopecia areata (AA), a cutaneous autoimmune disease characterized by hair loss, seen in Philadelphia, Pennsylvania [6].

## Materials and methods

The medical records of 439 pediatric patients with a diagnosis of AA between January 2015 and December 2017 were reviewed. A total of 96 children with 25-hydroxyvitamin D levels and no documented vitamin supplementation, chronic illness, or other autoimmune comorbidities other than AA were identified and included in the analysis [6]. Demographic data, Fitzpatrick skin type (I, II, III, IV, or V), medical history, vitamin D level, the month of blood collection (January-December), Severity of Alopecia Tool (SALT) score within three months of the assay, and most recent diagnosis (alopecia areata, alopecia universalis, or alopecia totalis) were recorded. Monthly UV index information from Philadelphia, PA (range 1-7) corresponding to the month of blood collection was also collected from World Weather Online [7]. The season of blood collection was evaluated as summer (June through August) versus non-summer. The race was evaluated as white versus non-white races. Vitamin D levels were classified as insufficient (<30 ng/ml) or sufficient (≥30 ng/ml) [8]. Univariate and multivariate logistic regression models were created to measure the relationship between these patient characteristics and vitamin D insufficiency. Statistical analyses were conducted using STATA/IC software (version 16, StataCorp LP, College Station, TX).

## Results

A total of 96 children met the inclusion criteria. Of the included patients, 60.4% had insufficient vitamin D levels, of which 38.2% were deficient. The mean age was nine years old, with a standard deviation (SD) of 4.4 years. The age range of the participants was 1-17 years old. The majority were female (64%) and white (48%) and had insufficient vitamin D levels (60%; mean=26.7 ng/ml, SD=7.6 ng/ml). We evaluated age, gender, Fitzpatrick skin type, and UV index at the month of blood collection in univariate and multivariate binary logistic regressions (Table 1). In multivariate analyses, higher Fitzpatrick skin type was associated with vitamin D insufficiency (OR: 2.40, 95% CI: 1.48, 3.89 for one unit increase), while higher UV index in the month of blood collection was inversely associated with vitamin D insufficiency (OR: 0.63, 95% CI: 0.48, 0.84 for one unit increase). Age and sex did not significantly predict vitamin D insufficiency, though age was borderline significant (OR: 1.10, 95% CI: 0.99-1.24 for a one-year increase). A separate multivariate analysis adjusting for age, sex, and UV index found that the non-white race was significantly associated with vitamin D insufficiency (OR: 4.22, 95% CI: 1.64-10.89) compared to white. A separate multivariate analysis adjusting for age, sex, and Fitzpatrick skin type found that the non-summer season of serum collection was associated with a significantly increased odds of vitamin D insufficiency (OR: 4.64, 95% CI: 1.39-15.44) compared to summer. Analyses were separated, thus, due to collinearity between race and Fitzpatrick skin type, as well as between UV index and season of serum collection.

**Table 1:** Odds ratio and 95% confidence interval (95% CI) of predictors of vitamin D deficiency among children with alopecia areata *Multivariate analysis was adjusted for age (continuous), sex (male or female), Fitzpatrick skin type (continuous), and UV index during the month of blood collection (continuous).

	N (total)	N (insufficient)	Univariate (95% CI)	Multivariate* (95% CI)
Age (continuous)	96	58	1.07 (0.98, 1.18)	1.10 (0.99, 1.24)
Sex	96	58		
Female	61	22	1.00 (reference)	1.00 (reference)
Male	35	36	1.18 (0.50, 2.76)	0.96 (0.33, 2.82)
Fitzpatrick skin type (continuous)	92	55	3.52 (1.40, 3.29)	2.40 (1.48, 3.89)
UV index (continuous)	95	57	0.69 (0.54, 0.88)	0.63 (0.48, 0.84)

A stepwise linear regression of vitamin D levels on the included covariates was also conducted. Higher Fitzpatrick skin type and age were inversely related to vitamin D levels (p<0.05), while UV index at the month of serum collection was positively related to vitamin D levels (p<0.05).

## Discussion

We found that non-White race, higher Fitzpatrick skin type, and non-summer seasons were significantly associated with increased odds of vitamin D insufficiency, while a higher monthly UV index was inversely associated with suboptimal vitamin D levels. This study contributes to the paucity of literature surrounding predictors of vitamin D levels in US children. One study of preschool children in New Zealand found that female gender, non‐European ethnicities, olive‐dark skin color, lack of vitamin D supplementation, having mothers with less than secondary‐school qualifications, and living in more deprived households were associated with insufficient vitamin D levels [3]. A similar study of Australian preschool children observed that the season of blood collection and having a mother born in Australia were significant predictors of serum vitamin D concentration. Age, sex, socio-economic status, body mass index (BMI), and dietary vitamin D supplement use were not related to vitamin D status [5]. Another study of Canadian Hutterite children between the ages of 3 and 15 found that age and latitude were negatively associated with serum vitamin D levels [4]. Consistent with some of these studies, we found that non-Caucasian ethnicity and season were predictors of low vitamin D levels; however, we did not observe a significant difference in odds between age or gender. While some of the studies evaluated seasons, none of the previous studies evaluated the monthly UV index in relation to vitamin D insufficiency.

Limitations of this study include a small sample size and the specificity of the cohort. Although average serum 25 hydroxy-vitamin D levels observed in AA patients were generally below the reported averages in healthy US pediatric populations (32.1-35.2 ng/mL), the frequency of deficiency in our sample (22% with vitamin D levels <20 ng/mL) was identical to that observed in a larger pediatric population in the US (22%) [9,10]. Consequently, we believe that these results may be potentially generalizable to demographically and geographically similar pediatric populations in the US, although our patients were not a random sample of the populations. Furthermore, the protocol for screening patients was not standardized and was often prompted by parental request. We also lacked information on some previously identified predictors of vitamin D insufficiency, such as BMI.

Our study also had several strengths. Because the study was conducted in a geographical area with four seasons, we were able to evaluate the impact of season and UV index. Moreover, our study subjects were racially diverse, with 52% identifying as non-white.

## Conclusions

In conclusion, we identified some predictors of vitamin D insufficiency in children and adolescents in Philadelphia, PA. Larger studies are warranted to replicate our findings and fully evaluate predictors of pediatric vitamin D deficiency in the US. In geographic regions with seasons, the role of the season may need to be taken into account in evaluating vitamin D levels. Varying diets across geographic regions of the US should also be considered in future national studies.

## References

[1] Guo J, Lovegrove JA, Givens DI (2019). A narrative review of the role of foods as dietary sources of vitamin D of ethnic minority populations with darker skin: the underestimated challenge. Nutrients.

[2] Misra M, Pacaud D, Petryk A, Collett-Solberg PF, Kappy M (2008). Vitamin D deficiency in children and its management: review of current knowledge and recommendations. Pediatrics.

[3] Cairncross CT, Stonehouse W, Conlon CA (2017). Predictors of vitamin D status in New Zealand preschool children. Matern Child Nutr.

[4] Science M, Maguire JL, Russell ML, Smieja M, Walter SD, Loeb M (2017). Prevalence and predictors of low serum 25-hydroxyvitamin D levels in rural Canadian children. Paediatr Child Health.

[5] Zhou SJ, Skeaff M, Makrides M, Gibson R (2015). Vitamin D status and its predictors among pre-school children in Adelaide. J Paediatr Child Health.

[6] Putterman E, Castelo-Soccio L (2018). Response to "Vitamin D deficiency in patients with alopecia areata: A systematic review and meta-analysis" and an investigation of vitamin D in pediatric patients. J Am Acad Dermatol.

[7] (2022). Providence weather forecast. https://www.worldweatheronline.com/providence-weather/rhode-island/us.aspx.

[8] Holick MF, Binkley NC, Bischoff-Ferrari HA (2011). Evaluation, treatment, and prevention of vitamin D deficiency: an Endocrine Society clinical practice guideline. J Clin Endocrinol Metab.

[9] Turer CB, Lin H, Flores G (2013). Prevalence of vitamin D deficiency among overweight and obese US children. Pediatrics.

[10] Saintonge S, Bang H, Gerber LM (2009). Implications of a new definition of vitamin D deficiency in a multiracial us adolescent population: the National Health and Nutrition Examination Survey III. Pediatrics.

